# Peptidic Antifreeze Materials: Prospects and Challenges

**DOI:** 10.3390/ijms20205149

**Published:** 2019-10-17

**Authors:** Romà Surís-Valls, Ilja K. Voets

**Affiliations:** Laboratory of Self-Organizing Soft Matter, Laboratory of Macro-Organic Chemistry, Department of Chemical Engineering and Chemistry & Institute for Complex Molecular Systems, Eindhoven University of Technology, Post Office Box 513, 5600 MD Eindhoven, The Netherlands; r.suris.valls@tue.nl

**Keywords:** antifreeze (glyco)proteins, peptide mimics, synthetic materials, ice recrystallization inhibition, antifreeze analogues, cryopreservation

## Abstract

Necessitated by the subzero temperatures and seasonal exposure to ice, various organisms have developed a remarkably effective means to survive the harsh climate of their natural habitats. Their ice-binding (glyco)proteins keep the nucleation and growth of ice crystals in check by recognizing and binding to specific ice crystal faces, which arrests further ice growth and inhibits ice recrystallization (IRI). Inspired by the success of this adaptive strategy, various approaches have been proposed over the past decades to engineer materials that harness these cryoprotective features. In this review we discuss the prospects and challenges associated with these advances focusing in particular on peptidic antifreeze materials both identical and akin to natural ice-binding proteins (IBPs). We address the latest advances in their design, synthesis, characterization and application in preservation of biologics and foods. Particular attention is devoted to insights in structure-activity relations culminating in the synthesis of de novo peptide analogues. These are sequences that resemble but are not identical to naturally occurring IBPs. We also draw attention to impactful developments in solid-phase peptide synthesis and ‘greener’ synthesis routes, which may aid to overcome one of the major bottlenecks in the translation of this technology: unavailability of large quantities of low-cost antifreeze materials with excellent IRI activity at (sub)micromolar concentrations.

## 1. Introduction

Ice-binding proteins (IBPs) comprise a unique class of biomacromolecular ice crystal growth modifiers that offer their host a means to thrive in extreme environmental niches. As unique binders of ice crystal planes, IBPs aid survival in sub-zero ice-laden habitats in various ways ranging from ice adhesion to stay put in a nutrient-rich environment [1] to inhibition of ice recrystallization [2], which protects biological tissues against mechanical damage [1,3]. Widely researched are the two IBP subclasses of antifreeze proteins (AFPs) and antifreeze glycopeptides (AFGPs), collectively referred to with the umbrella term AF(G)Ps. Binding of AF(G)Ps to ice creates a modest thermal hysteresis (TH) gap between a dose-dependent non-equilibrium melting and freezing point. In this TH gap, the proteins maintain fluids in an undercooled state wherein further growth of small, single ice crystals is arrested. This characteristic feature sets AF(G)Ps apart from all other compounds, including osmolytes and IBPs that bind ice but do not produce a TH gap as they depress the freezing and melting points in a far less efficient colligative manner. Inspired by the remarkable efficacy of AF(G)Ps to halt ice growth at (sub)micromolar concentrations, various approaches have been proposed over the past decades to emulate these cryoprotective features using tailored (bio)materials based on peptides [3,4,5], small molecules [6], inorganic compounds [7], and macromolecules [8,9,10]. In this review we discuss the prospects and challenges associated with this ambitious endeavor focusing especially on peptidic antifreeze materials both identical and similar to natural IBPs. Highlighting recent work in particular, we summarize the current state-of-the-art and review the latest advances in design, synthesis, characterization and potential applications in cryopreservation, food industry and construction. This includes new insights in structure-activity relations culminating in the synthesis of de novo peptide analogues of naturally occurring IBPs. Furthermore, we describe innovations in fluorenylmethyloxycarbonyl (Fmoc) based solid-phase peptide synthesis including the introduction of ′greener′ routes. Next we address interesting studies on translational efforts towards potent cryoprotectants, freeze-thaw mitigating additives to mortar, and other application area in recent work. Finally, we offer a perspective for future research of fundamental and applied interest.

## 2. Structure and Ice-Binding Sites of Antifreeze (Glyco) Proteins

IBPs expressed in microbes, plants, fish, algae and insects are both structurally and functionally diverse. To date, five biological functions have been characterized: freezing point depression [11], inhibition of ice recrystallization [12,13], modulation of ice nucleation [14], retention of liquid pockets [15], and ice adhesion [16]. In the following we briefly summarize the structural characteristics of selected AFPs which are glycosylated (AFGPs) and AFPs which are α-helical, rich in β-strands (β-clips, β-solenoids), lectin-like, and display polyproline II helices [1]. These inspire the design of (peptidic) IBP mimics, which often contain (a portion of) naturally occurring ice-binding sites and strive to emulate the three-dimensional folds found in native IBPs.

### 2.1. α-Helical AFPs

**Figure 1:**
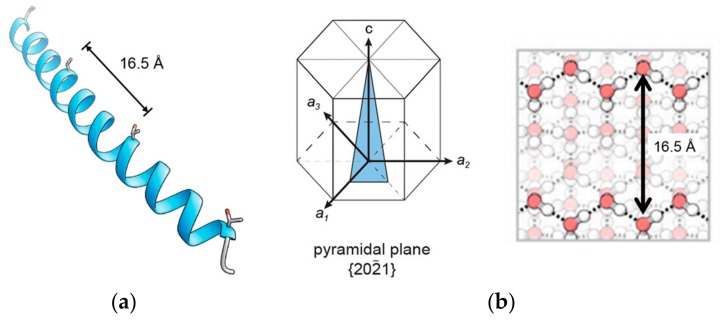
(**a**) Type I *wf*AFP from winter flounder (PDB:1WFA). The distance between threonine residues in *wf*AFP is 16.5 Å; (**b**) Representation of a pyramidal plane and the molecules that interact with the ice-binding site of *wf*AFP in a hexagonal ice crystal lattice with four lattice vectors indicated: the prism axis, c, which is normal to the basal plane, and the three vectors a_1_, a_2_, and a_3_, through alternate hexagonal points. Reprinted with permission from *Proc. Natl. Acad. Sci. USA* **2016**, *113*, 3740–3745. Copyright (2016) National Academy of Sciences [22].

Several species of fish including the winter flounder, yellowtail flounder and shorthorn sculpin express α-helical AFPs. These fish type I AFPs are typically present as a mixture of isoforms differing in antifreeze activity [17,18]. Widely investigated is the HPLC-6 isoform of the winter flounder type I AFP, in short *wf*AFP (Figure 1a). It has an α-helical structure consisting of 37 amino acids arranged in a sequence of three 11-amino acid repeats of threonine residues separated mainly by alanine residues [17,19]. The high alanine content (62%) contributes to the propensity of the protein to adopt an α-helical conformation [20]. This helical periodicity places all hydrophilic residues such as Thr on one face of the helix with a spacing of around 16.6 Å. Molecular modeling and docking studies showed that the opposite, Ala-rich face binds ice, suggesting that hydrophobic interactions are involved [17,18]. These results were supported by 13C solid-state NMR spectroscopy experiments, which revealed four times lower relaxation times for the methyl groups of alanine residues in the IBS compared to non-IBS alanine residues [21]. This decrease in relaxation time was attributed to the interaction between protons from water and methyl nuclei in alanine, suggesting interaction with the ice surface in the ice/water interface.

Computational studies showed that the hydroxyl groups of the four Thr residues in the *wf*AFP (HPLC 6 isoform) are aligned within the helical axis and the oxygen-oxygen spacing between hydroxyl groups is 16.1 Å, 16.0 Å and 16.2 Å, respectively [23]. This matches the oxygen-oxygen spacing on the pyramidal plane of ice, thereby favoring binding of the AFP to the pyramidal plane (Figure 1b).

### 2.2. β-Strand AFPs

**Figure 2:**
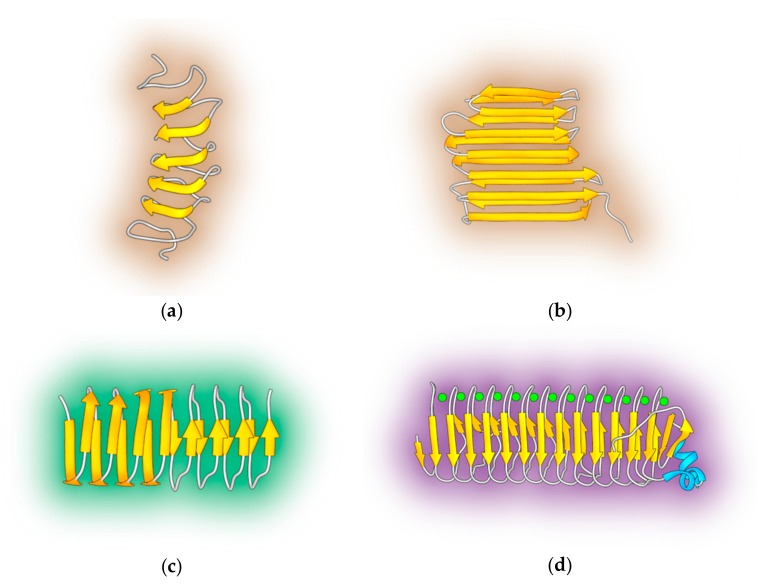
Overview of several AFPs with β-helix secondary structure. (**a**) *Tm*AFP (PDB:1L1I); (**b**) the insect protein *Ri*AFP (PDB:4DT5); (**c**) the ryegrass *lp*AFP (PDB:3ULT), and (**d**) the calcium-dependent Antarctic bacteria antifreeze protein *Mp*AFP (PDB:3P4G).

Fish AFPs do not contain β-helices, but the structural motif is abundant in AFPs from insects, bacteria and over-wintering plants. The beetle *Tenebrio molitor* has several isoforms of the protein *Tm*AFP [24], all of which contain several 12- or 13-amino acid repeats that organize into a right-handed β-helical structure (Figure 2a). Eight disulfide bridges in the 32 Å long β-helix augment its stability. 

The IBS consists of regular arrays of TXT motifs positioned on one side of the helix. Similarly, the spruce budworm *sbw*AFP contains repeating patterns of 15 amino acids organized in a β-helix structure with a triangular cross-section and arrays of TXT motifs at the flat IBS [25]. The display of ice-binding threonines is even larger in the larger insect AFP from the longhorn beetle *Rhagium inquisitor*. The *Ri*AFP (Figure 2b) IBS contains five internal repeats of a seven residue pattern TXTXTXT, wherein X is a small residue like alanine or threonine. The IBP of the Antarctic bacteria *Marinomonas primoryensis* also contains a right-handed β-helix [26]. The ice-binding domain is part of an impressively large, calcium-dependent adhesin with an overall molecular weight of 1.5 MDa. Five distinct regions with different functions (anchoring, extension, peptide recognition, sugar recognition and ice binding) have been identified in the whole construct, of which region IV is responsible for ice binding (Figure 2b). The AFP from ryegrass *Lolium perenne* (Figure 2c) folds as a left-handed β-helix with a length of 33 Å and width of 20 Å [27]. Interestingly, the β-helix structures from bacteria and insects are threonine-rich, but the β-helical coil of ryegrass is not. The *Lp*AFP IBS is composed of two seven-residue tandem repeats (XXNXVXG), wherein X is a residue with a polar side chains [27,28], therefore more hydrophilic than the threonine-rich IBS of insects and microbes.

### 2.3. Antifreeze Glycoproteins

**Figure 3:**
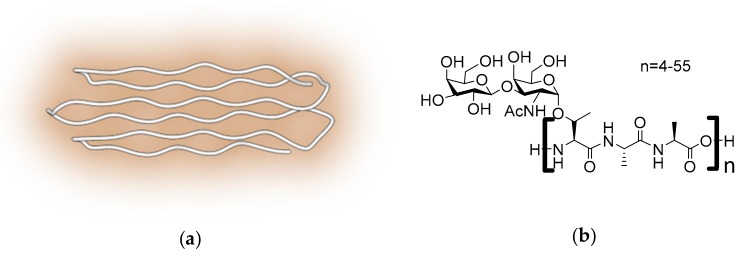
PPII helix containing AFPs and antifreeze glycoproteins. (**a**) Snow flea AFP (PDB:2PNE) showing six short type II polyproline helices; (**b**) Chemical structure of AFGPs from Antarctic notothenioids.

AFGPs are a group of eight structurally related glycoproteins present in the blood serum of Antarctic notothenioids and northern cod. These proteins consist of 4-55 tripeptide units of AAT that are *O*-glycosylated at the threonine side chains with the disaccharide β-D-galactosyl(1–3)-α-*N*-acetyl-D-galactosamine (Figure 3b). AFGPs exist as a mixture of compounds rather than as a single compound, where eight different isoforms have been isolated and categorized by electrophoretic techniques. AFGP_1_ is the largest glycoprotein and has a molecular weight of roughly 34 kDa. AFGP_8_ is the smallest of them with a molecular weight of 2.6 kDa (Figure 3b). Detailed conformational studies by circular dichroism (CD) and NMR spectroscopy did not reveal a well-defined three-dimensional structure for large AFGPs in solution. Presumably, AFGPs are rather flexible and dynamic and—akin to synthetic polymers—many configurations co-exist in solution. Simulations [29], CD spectroscopy and solid-state NMR on AFGP_8_, the smallest AFGP isoform, suggest that adopts PPII structures due to the discrete distribution of prolines in the amino acid sequence [30]. 

### 2.4. Polyproline II Containing AFPs

Type II Polyproline (PPII) helices are displayed in insect AFPs without α- nor β-helices, such as the snow flea AFP (Figure 3a). Molecular modeling studies indicated that the snow flea protein is organized into a bundle of six short PPII helices without any other secondary structure [31]. This PPII structure is due to a high glycine content and the presence of periodically distributed prolines throughout the amino acid sequence [32]. The predicted IBS is a flat hydrophobic surface which contains mainly small hydrophobic amino acids such as alanine, valine and thronine.

## 3. Ice-Binding and Activity

**Figure 4:**
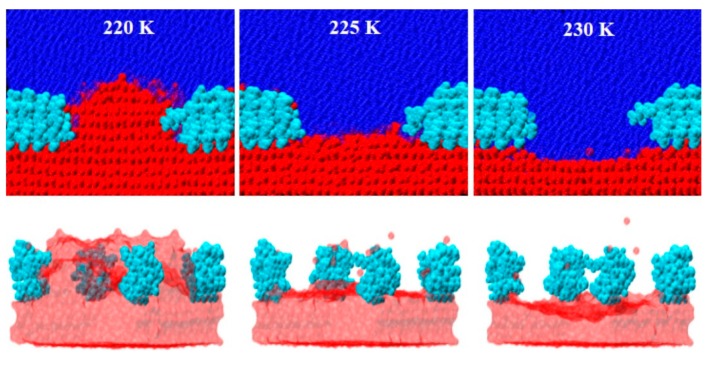
Atomistic molecular dynamics simulations on *Tm*AFP show inhibition of ice growth and melting below and above the equilibrium melting temperature, respectively, through the formation of convex and concave ice-water interfaces. Reprinted (adapted) with permission from Midya, U.S.; Bandyopadhyay, S. Operation of Kelvin Effect in the Activities of an Antifreeze Protein: A Molecular Dynamics Simulation Study. *J. Phys. Chem*. **2018**, *122*, 3079–3087. Copyright (2018) American Chemical Society [41].

Ice-binding is a prerequisite for all AF(G)Ps to function. Yet, the physico-chemical principles of the interaction between AF(G)Ps and ice remain elusive, as both experimental and theoretical studies of protein binding to ice are challenging. In recent years, computational studies [29,33,34,35,36,37] and microscopy experiments [38,39,40] have been particularly illuminating in this area. The simulations of Midya, and Bandyopadhyay [41] beautifully illustrate how AF(G)Ps can arrest growth via the Kelvin effect as originally proposed by Knight and DeVries [42,43]. Antifreeze proteins adsorb onto growing ice surfaces *via* their IBS. Growth ceases at the location of the bound AFPs, but continues in between the adsorbed proteins until the addition of water molecules to the convex surface is no longer energetically favorable. Ice growth is now completely halted and the freezing point depressed. Growth remains prohibited as long as the temperature remains above the non-equilibrium freezing point, which depends on the magnitude of the local curvature set by the spacing between bound proteins and the undercooling (Figure 4). In the following sections, we will briefly describe various assays tailored to probe the thermal hysteresis activity, ice recrystallization inhibition activity, and ice-plane affinity of ice-binding proteins.

### 3.1. Ice Plane Recognition

**Figure 5:**
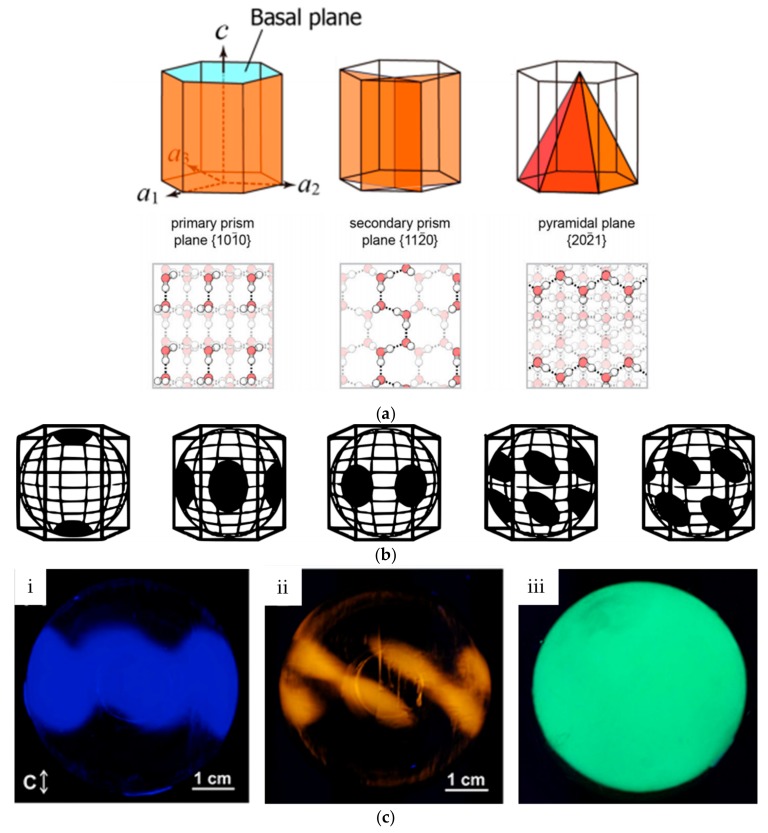
(**a**) Cartoon representation of specific ice crystal planes in the hexagonal ice lattice to which IBPs bind. The basal plane (normal to the c-axis) is shown in cyan. In orange are depicted the primary prism planes (left), the secondary prism planes (middle), and the pyramidal planes (right). Reprinted with permission from *Sci. Rep.* **2019**, *9*, 2212; (**b**) Fluorescence-based ice plane affinity (FIPA) experiments on a single ice crystal using fluorescently tagged AFPs to identify the planes the proteins attach to. The planes identified using this technique are (from left to right): basal plane, primary prism plane, secondary prism plane, pyramidal plane aligned with a-axes, pyramidal plane offset of a-axes. (**c**) FIPA experiments on (i) pacific-blue-labeled type III AFP, (ii) TRITC-labeled type I AFP, (iii) and GFP-tagged *Mp*AFP. Adapted from Basu, K., Garnham, C. P., Nishimiya, Y., Tsuda, S., Braslavsky, I., Davies, P. Determining the Ice-binding Planes of Antifreeze Proteins by Fluorescence-based Ice Plane Affinity. *J. Vis. Exp.* **2014**, *83*, e51185, doi:10.3791/51185 [44].

Ice-binding proteins binds ice, but not in the same manner. Preferential adsorption of IBPs onto specific sets of ice crystal planes (Figure 5a) in specific orientations can be monitored in ice-etching experiments [26,44,45,46] assisted by fluorescence detection in a so-called fluorescence-based ice plane affinity (FIPA) assay [28,44]. These yield characteristic ice-etching patterns upon adsorption of IBPs onto ice hemispheres, which are related to the specificities of IBPs for certain ice planes and binding orientations [44] (Figure 5b). AFGPs from the Antarctic notothenioids and northern cod bind to the primary prism plane (1010) [47]. Type III AFP from the ocean pout also binds to the primary prism (1010) plane, but in addition also recognize the pyramidal plane (2021) [48]. This pyramidal plane is also recognized by type I AFP from the winter flounder, whereas the shorthorn sculpin AFP adsorbs onto the (2110) secondary prism plane [49] (Figure 5c). Hyperactive proteins such as the insect protein *D*AFP-1 and the *Mp*AFP from the Antarctic bacterium *Marinomonas primoryensis* bind up to three different crystallographic planes. These proteins bind with high affinity to the basal plane (0001) and can also recognize both the primary and secondary prism (1120) [26,50]. 

### 3.2. Thermal Hysteresis

**Figure 6:**
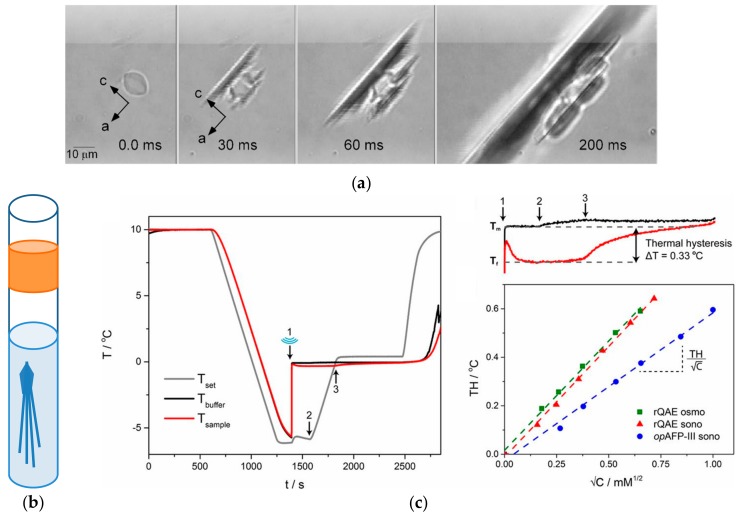
Different methodologies to quantify the thermal hysteresis (TH) activity of AF(G)P solutions. TH determined by (**a**) nanoliter osmometry (crystallographic a and c-axes are indicated) Adapted with permission from Bar, M.; Celik, Y.; Fass, D.; Braslavsky, I. Interactions of β-Helical Antifreeze Protein Mutants with Ice. *Crystal Growth & Design* **2008**, *8*, 2954–2963. Copyright 2008 American Chemical Society [58]. Sample cell for (**b**) the capillary method, in which samples are loaded with a capillary micropipette into the center of an oil-filled well or sealed in an oil-sealed capillary, respectively. Once a single ice crystal is obtained, the sample temperature is slowly decreased at a constant rate until the temperature of “burst”, which is the temperature at which explosive growth of the single ice crystal is observed. (**c**) Thermal hysteresis profile by sonocrystallization. A 1ml sample of AF(G)P solution is supercooled to −6 ^o^C after which a sonocrystallization pulse (100 ms) induces ice nucleation in the sample, which gives rise to a rapid temperature increase. The TH gap is calculated from the difference between the melting point and temperature of “burst”. Reprinted with permission from *Proc Natl Acad Sci USA* **2016**, *113*, 3740–3745. Copyright (2016) National Academy of Sciences [22].

Critical for the survival of freeze-avoidant fish and insects is the thermal hysteresis (TH) or antifreeze activity of antifreeze (glyco)proteins. Ice-binding of AF(G)Ps generates a so-called TH gap between a non-equilibrium freezing and melting point (*T*_f_, *T*_m_) in which nucleated ice crystals cease to grow. These blocks further growth of internalized ice in e.g., the intestinal tract of fish living in ice-laden waters. The magnitude of the generated TH gap (i.e., TH = *T*_m_ − *T*_f_) is strongly dependent on AF(G)P type and concentration [22]. The driving forces for ice-binding, the irreversibility of ice adsorption, and their relation to IBP activity remain a heavily debated topic [38,40,50]. A series of fluorescence microscopy experiments point towards quasi-permanent binding of AF(G)Ps to ice [40,51,52,53,54], which is considered essential for TH activity [55]. Pronounced freezing point depression has been attributed to basal-plane affinity [38], but this proposition has been contested by a recent discovery of IBPs derived from the microalgae *Fragilariopsis cylindrus*, which display moderate freezing point depression yet suppress basal plane ice growth [38]. Various experimental methodologies have been developed to accurately measure TH activity, including direct visualization of single ice crystals in 2–5 μL samples in a capillary technique (Figure 6b) [56] and in ~10 nL samples in nanoliter freezing point osmometry (Figure 6a). Alternatively, the freezing and melting points can be determined from the heat effects monitored directly in a non-invasive, automated sonocrystallization experiment of TH activity on 1 mL samples (Figure 6c) [22,57]. The larger sample volume is disadvantageous, but samples may be recovered for further experiments.

### 3.3. Ice Recrystallizaiton Inhibition

Ice-binding proteins in freeze-tolerant species such as overwintering plants inhibit recrystallization in e.g., plant apoplasts to prevent cell and tissue damage [3]. Ice recrystallization is a thermodynamically driven process wherein large crystals grow at the expense of smaller ones, thereby lowering the free energy of the system. Ice recrystallization inhibition (IRI) is assayed in various ways all of which rely on detection of ice crystal growth by microscopy. 

**Figure 7:**
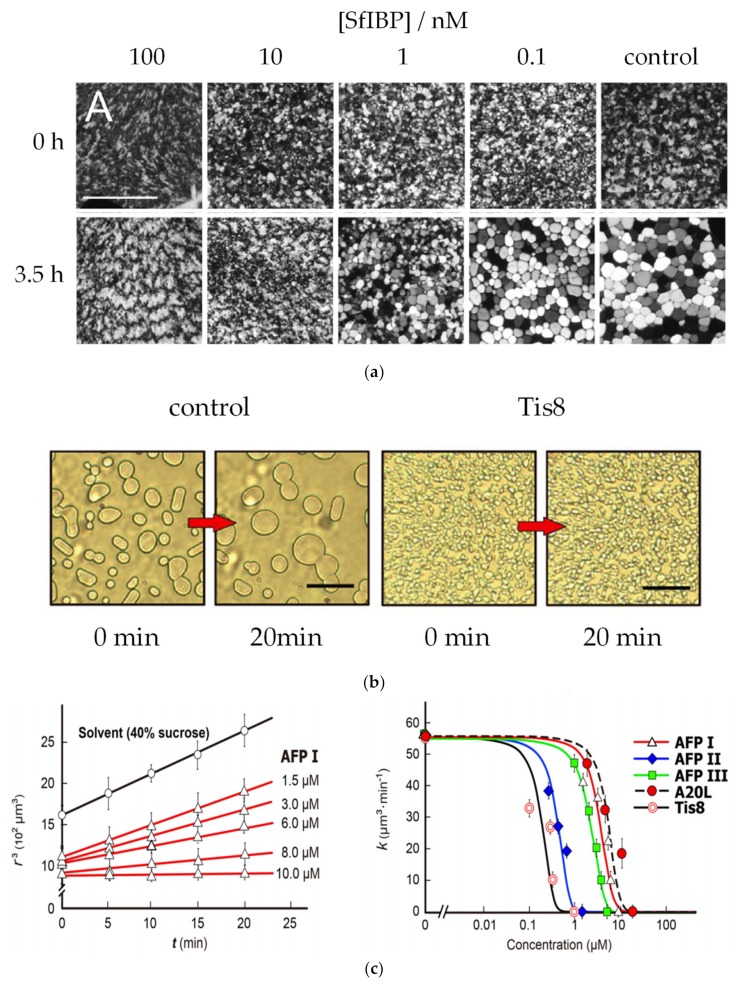
Overview of IRI assays. (**a**) Endpoint analysis of samples containing 100 nM of ice-binding proteins from *Spodoptera frugiperda* (*Sf*IBP) compared to a buffer as reference sample. Crystal growth is clearly visible in the reference sample, while ice crystals remain small at sufficiently high concentrations of the IRI active compounds. Reprinted with permission from Graham, L.A.; Agrawal, P.; Oleschuk, R.D.; Davies, P.L. High-capacity ice-recrystallization endpoint assay employing superhydrophobic coatings that is equivalent to the ‘splat’ assay. *Cryobiology* **2018**, *81*, 138–144 [59]. In a sucrose sandwich assay, (**b**) migratory ice recrystallization is monitored. (**c**) To rank IRI active compounds, dose-dependent ice crystal growth rates k are determined from the temporal evolution of the cubic number average mean radius (r^3^) for a relevant range of concentrations. Reprinted with permission from *Sci. Rep.* **2019**, *9*, 2212 [13]. The compound that decelerates ice growth at the lowest protein concentration is regarded as most active (here Tis8).

In a classical ′splat assay′ (Figure 7a), the time-evolution of the mean (largest) grain size (MGS or MLGS) is monitored in a thin polycrystalline wafer of ice, which is kept for several hours at a fixed annealing temperature. Gibson and coworkers proposed that the relative reduction in MLGS by an inhibitor compared to a negative control is a useful metric to distinguish between potent, moderate, and poor inhibitors with high, modest and low/no IRI activity [60]. Davies et al. proposed an endpoint analysis where the lowest AF(G)P concentration shows IRI activity [59], wherein up to 12 samples of a series of dilution are analyzed at the same time. Samples are placed side-by-side into wells etched from a sapphire crystal slide (Figure 6a). After snap-freezing in liquid nitrogen, the multi-crystalline wafer is observed by optical microscopy to monitor the grain size over time. Quantitative IRI assays use low molecular weight solutes such as salts or sucrose to accelerate recrystallization processes and ensure a sufficiently large non-frozen fraction to avoid false positives. In ′sandwich assays′ of IRI activity microliter samples ′sandwiched′ between two coverslips are observed of AF(G)Ps in 20–45 wt% sucrose solutions with ice crystal volume fractions < 0.3 thus focusing on migratory recrystallization (Figure 7b). Quantification is done using automated image analysis to extract the time-evolution of the cubic number average mean radius <*R*_n_>^3^ from the micrographs [61,62], which is analyzed in the framework of the Lifshitz, Slyozov and Wagner (LSW) theory for Oswald ripening (Figure 6c) [61]. 

## 4. Production of AF(G)Ps and Synthetic Analogues: Design and Chemical Synthesis Approaches

Since the pioneering experiments of DeVries and Wohlshlag on AFGPs purified from blood samples of Notothenioids fishes collected during Antarctic expeditions [63], the field has greatly benefited from various innovative technologies that made it possible to identify, isolate and produce AF(G)Ps in research laboratories [45,64,65,66]. In 1993 Chao, Davies, Skyes and Sönnichsen reported the first successful recombinant synthesis of the fish type III AFP from the eel pout *Macrozoarces americanus* in *E. coli*, which has since become the *Drosophila melanogaster* of the AFP field [67,68]. Importantly, recombinant synthesis not only improved AFP availability [17,69], but it also enabled new mechanistic studies on structure-function relations via site-directed mutagenesis [62,70]. Similarly, solid-phase peptide synthesis (SPPS) made fish type I AFPs more available to the field and facilitated mechanistic studies relating the importance of protein helicity. Inspired by native IBPs, various classes of IBP mimics including peptidic antifreezes have been developed aiming to e.g., enhance thermal hysteresis activity [71,72,73], produce IRI-active materials without TH activity [60,74], and manufacture at low cost in high yield cryoprotectants for cryopreservation of biologics [10,75,76,77].

Fmoc-based solid-phase peptide synthesis (SPPS) made a wide variety of peptidic antifreezes, such as cyclic peptides and glycopeptides [78,79,80], synthetically accessible as it enables the selective protection of specific functional groups while others remain accessible to react. In the following we distinguish and discuss two categories: molecular analogues and de novo peptide antifreezes (Table 1). Molecular analogues are peptides based on the native sequence of natural AF(G)Ps including chemically synthesized native AF(G)Ps and mutants thereof. De novo peptide antifreezes are non-native compounds inspired by naturally occurring IBPs, such as peptides with non-canonical amino acids, D-amino acids, and/or with side-chains conjugated via non-natural chemical links such as C-linked oligosaccharides and triazoles. 

### 4.1. Molecular Analogues

To date, chemical synthesis strategies have focused primarily on the smallest IBPs with a highly repetitive amino acid sequence and a relatively simple secondary structure. These are type I AFPs like *wf*AFP and AFGPs like AFGP_8_ from polar fishes, which are amenable to both solution phase synthesis and solid-phase peptide synthesis (SPPS). Chemical ligation has been employed to produce larger insect AFPs based on the beetle *Dendroides canadensis* from smaller fragments produced by SPPS.

To shed light on the relative importance of hydrophobicity and hydrogen bonding for ice-binding, various *wf*AFP mutants have been prepared by SPPS, wherein one or more threonine residues in the IBS carrying both OH and CH_3_ functionalities have been replaced by amino acids with either pendant OH groups (serine) or CH_3_ groups (valine) [69,81]. The more threonines were substituted by serines, the more TH activity was lost, ranging from 20% loss for 1 substitution (regardless of the position changed) to 100% loss for either 3 or 4 substitutions [73]. Substituting 2 out of 4 threonines for serine residues also resulted in a complete loss of TH activity if the remaining threonine residues were not close to one another. Substitution of all 4 threonines by valine did not result in a complete loss of activity, instead, 30% of the TH gap was retained [81]. Collectively, these results suggested that both functionalities are pivotal not only the hydroxyl group. Thus, hydrogen bonding is not the sole driving force for ice-binding of *wf*AFP. The methyl functionalities may bind inside the holes in the middle of six-membered water rings in the ice lattice, as observed for methyl groups of specific hydrophobic amino acids including Thr-18 in the IBS of ocean pout type III AFP [82]. Molecular dynamics simulations on *Tm*AFP reveal a distinct role for both hydrophobic and hydrogen-bonding groups in its IBS. Amongst other relevant actions, the methyl groups stabilize the anchored clathrate-like waters at the IBS and the hydroxyl functionalities dock it onto ice [83]. Another study demonstrates that IBS flatness is like IBS hydrophobicity prerequisite to maintain the thermal hysteresis activity of various AFPs. Substitution of the small hydrophobic amino acid alanine to the bulky hydrophobic amino acid leucine in the middle of the IBS of *wf*AFP led to the complete loss of thermal hysteresis activity in an A17L-*wf*AFP mutant [69].

**Figure 8:**
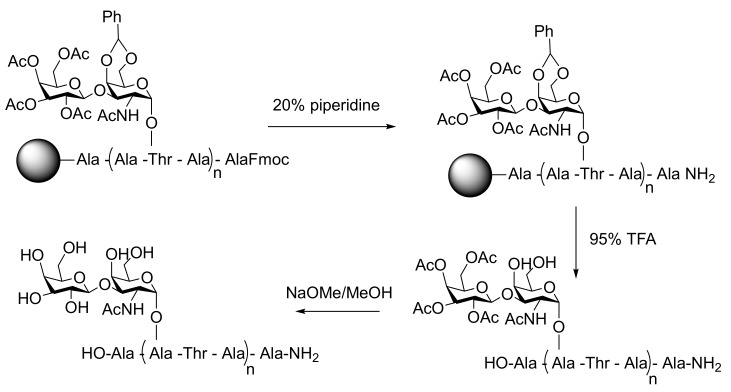
Solid-phase peptide synthesis of natural AFGPs. Acetyl groups protect the hydroxyl groups from the carbohydrate moiety attached to Thr used in the synthesis of the peptides. After the synthesis is finished, the glycopeptide is cleaved from the resin using strong acidic conditions. Finally, the protecting groups from the sugar are removed using sodium methoxide to obtain a chemically made AFGP.

Antifreeze glycoproteins are notoriously difficult to synthesize and characterize, but the challenge has been addressed successfully by several groups (Figure 8). Nishimura developed a synthetic route to natural AFGPs using solution phase polymerization of unprotected AAT glycopeptides in the presence of the initiator diphenylphosphorylazide (DPPA) [84]. To improve the yield by reducing steric hindrance, the AAT building blocks were later substituted by ATA [85]. In an attempt to obtain larger AFGP analogues resembling the native proteins, DPPA was substituted by the coupling reagent 4-(4-6-dimethoxy-1,3,5-triazin-2-yl)-4-methylmorpholinium (DMTMM) [86]. With this approach, polydisperse AFGP mimics were obtained with a weight average molecular weight of 7 kDa. The synthesized AFGP analogues were able to shape ice crystals at −0.2 °C. Several alternative approaches were developed to overcome the dispersity in sequence and length of the AFGP analogues prepared by solution phase polymerization. To this end, Wilkingson [87] and Tseng [87,88] first prepared oligomers with a well-defined sequence and length by SPPS employing Fmoc-protected amino acids. In a following step, these were coupled using benzotriazole-1-yloxytris (pyrrolidino)phosphonium (PyBOP) hexafluorophosphate [87,88]. Peptides with as little as two ATA repeats were found to be TH active [87]. Activity increased significantly with increasing chain length up to 5 repeats [89]. However, no further increase was observed among synthetic AFGPs with larger number of repeats. By contrast, native AFGPs comprising 50 repeats were found twice as TH active as native AFGPs with 5 repeats [90]. 

The largest chemically synthesized AFPs were based on the structure of the beetle *Dendroides canadensis* AFP [91]. First, several peptide fragments containing the Cys residues essential for the stability and secondary structure of *Dc*AFP were synthesized by SPPS. Next, the formation of disulphide bridges was promoted to ensure correct folding of the protein, which was confirmed by ^1^H-NMR. After Cys-Cys bridge formation, the different fragments were connected by chemical ligation in a step-wise fashion to synthesize the native protein. The same strategy was used to create a non-natural variant with lactam bridges instead of disulphide bridges upon substitution of the cysteine residues with lysine and aspartic acids. Importantly, this approach did not only improve the synthetic yield and purity, but the novel AFPs were also more stable than their natural disulphide bridge containing counterparts. The thermal hysteresis activity of the two constructs was similar, with TH values at a protein concentration of 10 mM between 0.1 and 0.35 °C [91].

### 4.2. de Novo Design and Synthesis

De novo antifreeze analogues offer unprecedented means to advance our understanding of the working mechanism of ice-binding proteins and may be prepared in a more straightforward and less time-consuming manner than native AFPs. Different orthogonal chemical ligations not present in natural AF(G)Ps have been investigated to improve synthetic accessibility and probe whether it impacts antifreeze activity. Constructs varying considerably in composition and architecture from native AFGPs have been investigated to identify the minimal requirements for activity and the impact of various structural elements, such as secondary folds, tethered sugars, and free N- and C-termini.

Inspired by type I AFPs, Wierzbicki and coworkers prepared a series of de novo constructs by SPPS comprising 43 amino acid long polypeptides with repetitive sequences of alanine and lysine residues [92]. Like *wf*AFP, these constructs had a high content of Ala facilitating the formation of an α-helical fold. Unlike *wf*AFP, these constructs were devoid of the threonine residues supposedly essential for ice binding. Instead, the constructs contained lysine residues to improve solubility in water. Surprisingly, despite the absence of Thr, the peptidic constructs exhibited thermal hysteresis activity, albeit low with a modest TH of 0.3 °C at 50 mM in 0.1 M ammonium bicarbonate buffer [93]. Glycine rich snow flea AFP is not available in large quantities, which hampers detailed mechanistic investigations. Kent and coworkers synthesized D-*sf*AFP, the right-handed amino acid sequence by employing chemical ligations. The sequential ligation was done with N-terminal cysteines incorporated as 1,3-thiazolidine-4-R-carboxylic acid (Thz) and a thioester at the C-terminal [32]. Aiming to prepare peptidic antifreezes that are not susceptible to acidic, basic or enzymatic hydrolysis, Ben′s group synthesized various C-linked glycoconjugates. In these AFGP-inspired analogues, the natural AAT repeat was replaced by GGK to tether by C-linked glycosylation a variety of sugar moieties.

**Figure 9:**
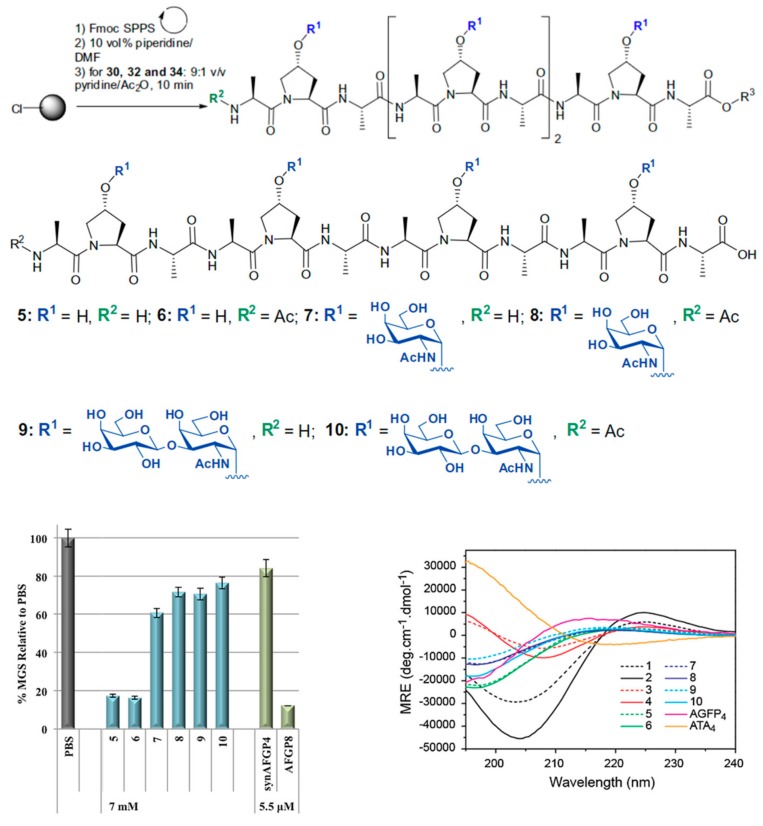
Synthesis scheme of polyproline based antifreeze peptides. PPII structures are built by synthesizing and modifying peptides containing high amounts of prolines in the sequence. The amino group of prolines can be further functionalized to accommodate moieties such as carbohydrates. PPII structures were confirmed by CD spectroscopy. Reprinted with permission from *Bioorg. Med. Chem.* **2013**, *21*, 3569–3581 [4].

Proline is unique amongst the canonical amino acids in that it has no amide N–H. Consequentially, polyproline cannot form intramolecular hydrogen bonds. In combination with the pyrrole functionality, this makes the polypeptide water-soluble and quite hydrophobic at the same time. Graham and coworkers hypothesized that polyproline helices can be considered the minimal AF(G)P mimic due to their implicit amphiphilicity. To demonstrate that, they performed a series of experiments with polyproline constructs of different length and stereochemistry [3]. These polyproline constructs showed mild IRI activity since they reduced the mean largest grain size by 50% at concentrations around 15 mM. In another study, a set of different PPII helices were synthesized using SPPS. In this case, peptide sequences resembled AFGP sequences wherein each threonine was replaced by hydroxyproline (Hyp) (Figure 9). The investigators functionalized the hydroxyl group from the Hyp residues with the carbohydrate present in natural AFGPs. Surprisingly, none of the peptides with the carbohydrates showed either TH activity, IRI activity or ice shaping. Peptides without carbohydrates had IRI activity since they were able to reduce the mean larger grain size of ice crystals by more than 80% compared to the PBS control. Moreover, the N-acetylated peptide induced some ice-shaping [3]. CD spectroscopy studies confirmed the PPII structure of both glycosylated and non-glycosylated compounds.

**Figure 10:**
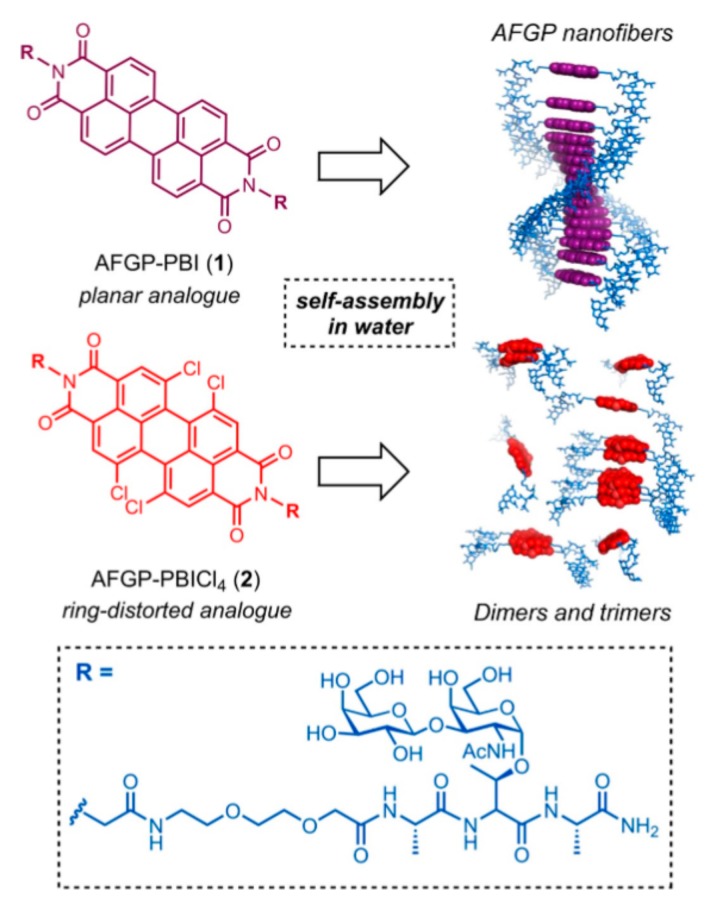
Scheme of the self-assembly induced by perylene bisimides and synthetic structure of the AFGP analogues attached. Reprinted with permission from *Chemistry* **2018**, *24*, 7834–7839 [74].

Drori and coworkers recently investigated the activity of self-assembling organic dyes that display specific functional groups in a periodic manner resembling the periodic display of functional groups in IBPs like AFGPs. The organic dye Safranine O was able to show TH activity of −0.32 °C at a concentration of 28 mM, a value comparable to AFGP8 (the smallest isoform) [6]. Moreover, IRI activity tests through ′sucrose sandwich′ assays demonstrated that Safranine O is able to inhibit ice recrystallization at a concentration of 4.2 mM. Based on this idea, Robert Ben′s group used perylene bisimides (PBI), a well-studied polyaromatic dye, to assemble one-dimensional aggregates displaying ice-binding motifs through supramolecular interactions and π-π stacking between subsequent PBIs (Figure 10). A regular PBI and a tetrachloro derivative were coupled to an AFGP analogue with modest IRI activity (80% MGS in splat assay) aiming to study the possible enhancement of IRI activity upon stacking of multiple short AFGP analogues [74]. PBI conjugates assembled into micron sized fiber-like structures at μM concentrations and inhibited ice recrystallization at 20 mM. Interestingly, neither of the constructs exhibit TH presumably due to a rather low affinity of the constructs for ice.

### 4.3. Cyclic Peptide Analogues

To test if the amino and carboxy group of AFGPs play a role in ice binding, Hachisu and collaborators prepared hexa-, nona- and dodecacyclic AFGP analogues. 2, 3 or 4 ATA repeats with the disaccharide β-D-galactosyl(1-3)-α-*N*-acetyl-D-galactosamine attached to the threonine were coupled using liquid phase peptide synthesis. To cyclize the linear peptides, the solution was diluted 100 times and fresh coupling reagent was added to the mixture. Interestingly, these cyclic analogs showed ice shaping properties and TH activity similar to their linear counterparts [85]. The TH activity was independent of the molecular weight of the AFGP analogue and a comparison in performance between the cyclic and linear peptides did not reveal any trend in TH activity. The retention of TH activity in cyclic AFGPs indicated that C- and N-termini are not essential to show thermal hysteresis. Brotzakis et al. studied the possibility of creating peptide nanotubes based in self-assembling cyclic octapeptides as putative ice-binding materials [98]. 

Their design was based in the insect antifreeze protein *Tm*AFP, which presents a β-helical rich structure where threonine amino acids are organized in a regular array of TXT. Previous experiments and simulations showed how cyclic peptides of alternating L- and D- amino acids self-assemble into nanotubes under various conditions [99,100,101]. Simulations revealed that nanotubes formed from octapeptides containing the motif TaT had a C^α^-C^α^ intramolecular distance of 7.04 Å, and a C^α^-C^α^ intermolecular of 4.88 Å. These values are close to the values shown for *Tm*AFP (7.35 Å and 4.5 Å, respectively) and the distances of hexagonal ice (7.356 Å and 4.518 Å). Molecular dynamics (MD) simulations also revealed a flat surface in the Thr-ala-Thr region, where on average more than one water interacts with the hydroxyl groups of the threonine residues through H-bonding.

### 4.4. Classifying Technologies to Aid de Novo Design

Virtual screening techniques, such as the basic local alignment search tool (BLAST) and quantitative structure-activity relationship (QSAR) models, have become an important tool in drug discovery [102,103] aiding the screening of large libraries of small molecules with the objective to find new compounds with therapeutic potential. These tools may speed up the drug discovery process and save costs through reducing the number of tested candidates. Several recent studies demonstrated that screening technologies also have the potential to discriminate between AFP and non-AFP sequences [104], to predict the activity of AFPs [22], and to inform efforts to design and synthesize *de novo* antifreezes [67,102]. 

Yang and coworkers compiled an effective predictor of new AF(G)Ps based on protein similarity using BLAST. A training dataset and an independent dataset consisting of 481 AFPs and 9,193 non-AFPs were used to create the basis of the predictor. These datasets contained all the diverse primary sequences and structures of known AF(G)Ps. The predictor used features of the AFP such as functional domain, the evolutionary conservation of these domains across proteins of the same family and statistical cross-validation to classify the best protein candidates that may show TH [105].

Ben′s group used a set of 124 previously synthesized compounds with known IRI activities to calibrate a 3D-QSAR model [66]. Input to train the 3D-QSAR model comprised of measured IRI activities and modelled three-dimensional structures, which were optimized by Monte-Carlo to identify the lowest energy conformations. On top of that, the team used an independent alignment of the 3D-descrpitors to correlate molecular surface curvature and electrostatical potential. The QSAR model was then used to prescreen a small library of novel putative IRI active compounds, and predicted that 11 small molecules in the library would be IRI active. Nine out of the eleven (82%) predicted compounds did reduce the main grain size in splat assays at concentrations between 11 and 22 mM.

Kozuch and collaborators combined molecular dynamics of water molecules near an AFP surface and geometric structure of the IBS with neural networks aiming to predict the magnitude of the thermal hysteresis gap of AFPs [106]. To build the predictive model, the team used 17 AFP structures desposited in the Protein Data Bank and their corresponding TH activity as a function of AFP concentration, *c*_AFP_. In addition, 5 proteins that show no TH but have exposed planar surfaces and/or secondary structures similar to AFPs were used as a negative control set. The generated model successfully predicted the antifreeze activity of known AFPs not included in the training set based on the assumption of a linear relation between TH activity and √*c*_AFP_ [22]. The deviation between predicted and experimental activity values was ≤0.19 °C.

## 5. Applications of AF(G)Ps and Analogues 

Applied interest in IBPs sparked soon after the discovery of the first AFGPs approximately 50 years ago and has continued to blossom in recent years into a highly active area of IBP research. This is because the protective properties displayed by IBPs are not only interesting for biomedical fields such as cryopreservation and cryosurgery [12,64,75,76,77,107], but may also mitigate freeze-thaw damage in frozen foods [108,109,110], civil engineering [111] and energy applications [112].

**Figure 11:**
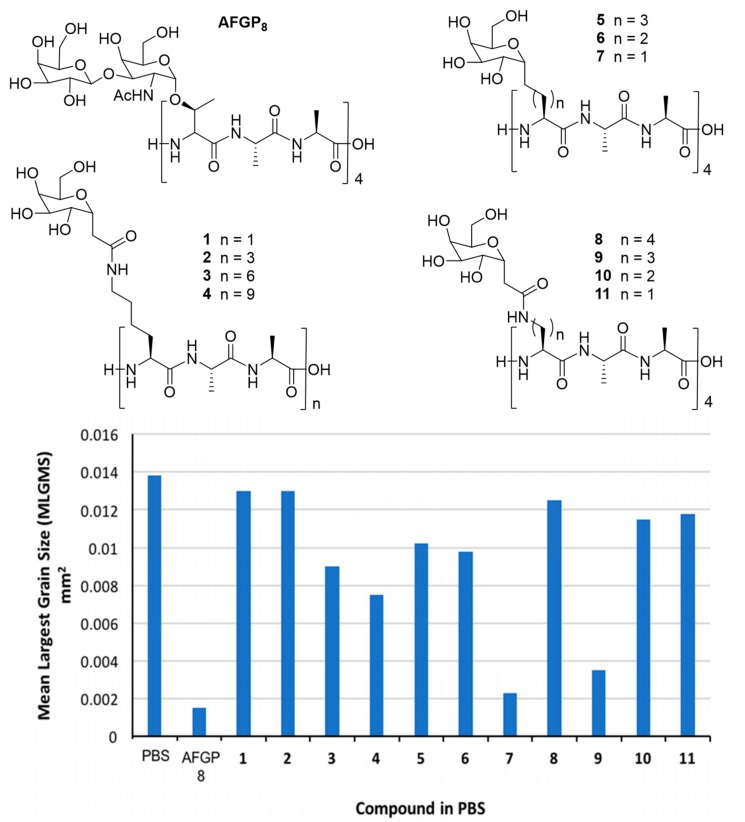
Set of different C-linked AFGP analogues synthesized using SPPS. The IRI activity of the different constructs was tested using the splat assay. Compound 7 shows similar potency in inhibiting ice recrystallization as the smallest AFGP (AFGP_8_). Reprinted with permission of *Pept. Sci.* **2019**, *111* [76].

Biomedical application of AF(G)Ps and their mimics is amongst the most active areas of IBP research. The interested reader is referred to recent reviews on the matter addressing in particular polymeric mimics [113], peptidic analogues [5] and native AF(G)Ps [114]. The potential of de novo peptidic antifreezes in this context remains largely uninvestigated [76]. Authors tested whether C-linked AFGPs synthesized by SPPS hold promise in cryopreservation (Figure 11). Compounds 7 and 9 increased cell viability after freeze-thawing at a low content of DMSO (10%). Moreover, the synthetic analogues showed no degree of toxicity when tested for 20 h at 37 °C in contact with WRL68 embryonic liver cells.

Starch, emulsifiers, proteins, and various other hydrocolloids are routinely used to maintain and stabilize the structure of food networks and emulsions. Likewise, AFPs and their analogues have been added to frozen foods to mitigate freeze-thaw induced alterations in texture and associated sensory properties. Peng and co-workers developed a food-grade expression and fermentation system based in protein expression in *Lactococcus lactis* for the production of large amounts of type I AFP. The lyophilized crude AFP was used to treat meat and dough to improve the post freeze-thaw structure, taste and composition. Frozen meat treated with type I AFP suffered less from protein loss and scored higher values in a sensory evaluation of juiciness. Dough treated with a crude lyophilized powder of type I AFPs showed better fermentation capacity than non-treated frozen dough [109]. Hydrolyzates of collagen from pigskin improved the texture of bread produced from frozen dough [115]. The loss of specific volume over 6 freeze-thaw cycles was reduced from 10% to 25%. AFPs from carrot homogenates ameliorated the cooking properties of frozen white salted noodles [110]. Treated noodles absorbed more water and lost less solid material (both proteins and carbohydrates) during cooking. Scanning electron micrographs (SEM) of the microstructure suggested that this is because AFPs help protect the integrity of the gluten network. Small peptides based on the amino acid sequence of *Dc*AFP altered the structure, cooking properties, and texture of freeze-thawed carrots [116]. Drip loss decreased with increasing soaking time prior to freeze-thawing. Exposure to the *Dc*AFP analogues for 7 days reduced drip-loss 5-fold. Freezing degraded the texture of both treated and untreated samples relative to the fresh vegetable, but not to the same extent. AFP-treated samples scored considerably better in the two key texture parameters for consumer preference: hardness and firmness. A comparative analysis by cryo-SEM revealed the microstructural changes underlying these positive effects. Due to the growth of larger (intracellular) ice crystals in the untreated samples, these display larger and more intercellular spaces, as well considerable cell membrane and cell wall deterioration. 

## 6. Prospects and Challenges 

Materials and tools have become available to elucidate the structure-function relations of the broad spectrum of structurally and functionally diverse ice-binding proteins that have been discovered. Long-standing open questions related to the working mechanism of ice-binding proteins may now be addressed both computationally and experimentally. Computationally guided design approaches and further optimization of synthetic strategies will facilitate further development of novel IBP analogues. Various ice-binding motifs may be incorporated into a single analogue to maximize efficacy. Assays of e.g. ice recrystallization activity could be further standardized to facilitate a fair comparison between the activiy of diverse classes of IBPs and their analogues. We also anticipate more in-depth studies on the application of IBPs and analogues in non-native environments geared towards an improved understanding of the factors that impact performance, so as to inform the selection of an appropriate IBP and/or the modular design of a suitable analogue. For example, we have recently investigated the impact of salts which are commonly present in cryopreservation media on the ice recrystallization activity of native AF(G)Ps. Interestingly, the study revealed that sodium borate has little impact on IRI-activity, while it dramatically reduced TH activity [12]. These findings suggest it is worthwhile investigating whether the salt composition of AF(G)P-containing cryopreservation media can be tuned to maximize the efficacy of AF(G)P on e.g., cell survival.

Synthetic approaches to produce peptidic antifreezes by solid-phase peptide synthesis can be further optimized to maximize yield, purity, and time-efficiency without generating large amounts of toxic waste, which constitute an environmental burden and health risk as chronic diseases may arise upon continued exposure. This motivated various successful attempts to adapt current protocols to utilize more environmentally benign solvents and coupling agents. Aiming to find a greener and safer alternative for DMF, McMillan and collaborators studied the effect of various solvents on amide bond-forming reactions [117]. Their results suggest that dimethylcarbonate (DMC) and 2-methyl tetrahydrofuran (2-MeTHF) can replace DMF and dichloromethane (CH_2_Cl_2_). Albericio´s team substituted DMF by THF or acetonitrile (MeCN) [118]. Synthesis in THF or MeCN diminished the degree of racemization of the tripeptide GCF, while the amide bond formation in THF or MeCN remained the same as amide bond formation in DMF. Strikingly, SPPS of hindered pentapeptides with the fungal amino acid 2-aminoisobutiric acid (Aib) resulted in higher synthesis yields (ranging from 2 to 4-fold increase) when THF or MeCN were used as solvents instead of DMF. Aiming to employ less hazardous coupling reagents, many researchers used COMU, OxymaPure and K Oxyma to suppress racemization instead of HCTU and HATU with excellent results [119,120]. Third-generation coupling reagents such as COMU are the most appealing as they are proven safer, give high yields, and disfavor racemization [120]. In sum, revisited peptide synthesis protocols must retain the simplicity and reaction yields from previous protocols, while implementing improved solvents and coupling reagents. Furthermore, they should be inexpensive for academic and industrial purposes and applicable to various chemical modifications, including cyclization and N-methylation, to be commercially interesting. The approaches discussed in the above have yet to be examined for amenability to long sequences and aggregation-prone peptides.

## 7. Concluding Remarks

Roughly 50 years since the first experiments on freezing-point depression by (glyco)proteins, the field of ice-binding proteins continues to flourish. The continued involvement of researchers from various backgrounds has stimulated scientific discussions, generated new research directions, and advanced both fundamental insight and efforts targeting utilization of antifreezes in various applications. Herein we have reviewed recent progress in IBP research focusing on peptidic antifreezes. We addressed their design, synthesis, characterization and application in preservation of biologics and foods. The widespread availability of diverse classes of IBPs, state-of-the-art experimental tools and computational methods may culminate in the near future in the elucidation of structure-activity relations aided by new developments in analogue synthesis, computational studies by molecular dynamics and quantum chemical calculations, and single-molecule experimentation. Bioinspired molecular designs of novel compounds will offer new insights into molecular recognition, as well as the relation between TH and IRI activity. The structural diversity of native IBPs suggests that it is possible to design a platform of new antifreeze materials varying in (amino acid) composition and higher order structure. Multiple putative ice-binding sites may also be combined into a single mimic aiming to bind to various ice crystal planes and enhance activity. Activity geared towards applications, especially in biomedicine, will continue to grow. We anticipate that the implementation of increasingly systematic and multidisciplinary approaches will bear fruit and ultimately deliver on the promise of IBPs as cryoprotectants in non-native environments.

## Figures and Tables

**Table 1:** Overview of studies on peptidic antifreezes listing compound type, synthesis method, activity assay type and protein concentration [A].

	AF(G)P Type	Synthesis Method	Activity Assay	[A] (mg mL^−1^ and/or μM)	Ref.
Molecular analogues	I	SPPS of mutant *wf*AFPs (A17L and A20L) substituting the alanine by leucine	TH	1, 2, 4 and 8 mg mL^−1^	[69]
	I	SPPS of analogues substituting threonine (T) residues by serine (S) or valine (V)	TH	0.5, 1, 1.5, 2, 4, 5.5 and 7 mM	[81]
	AFGP_8_	In-solution coupling of ATA building blocks with DPPA	TH	10 mg mL^−1^	[85]
	AFGP_8_	In-solution coupling of ATA building blocks using DPPA, DMTMM and IIDQ	TH	10 mg mL^−1^	[86]
	AFGP_2_ and AFGP_8_	Fmoc SPPS of AFGP building blocks, desulfurization of Thz and Cys residues	TH	5 and 10 mg mL^−1^	[87]
	AFGP_8_	Fmoc SPPS coupling with DCC/4-DMAP and PyBOP	Not tested		[88]
	AFGP_8_	SPPS of AAT building blocks with DPPA resulting in native AFGP molecular analogue	Not tested		[84]
	AFGP_8_	Automated Fmoc SPPS at 40 °C using TBTU as coupling reagent	TH IRI	30 and 40 mg mL^−1^ 0.5 μg mL^−1^	[94]
	*Dc*AFP	SPPS of peptide fragments of DcAFP chemically ligated afterwards	TH	10 mM	[91]
de novo Analogues	polyAAK	SPPS of type I analogues based on lysine instead of threonine	TH	from 25 to 250 mg mL^−1^	[92]
	polyAAK		TH	23 and 31 mM	[93]
	PPII	SPPS	Cryopres IRI	200 mM 20 mg mL^−1^	[3]
	PPII	SPPS of Hydroxyproline peptides functionalized with disaccharides	TH IRI	10 mg mL^−1^ 7 mM	[4]
	AFGP	SPPS synthesis of AFGP analogues attached to supramolecular organic dyes	TH IRI	22 mM	[74]
	AFGP_8_	Cyclized AFGP analogues synthetized by SPPS using DPPA	TH	10 mg mL^−1^	[85]
	AFGP_8_	SPPS of C-linked AFGPs to avoid O-linked degradation of disaccharides	Cyropres	From 1 to 5 mg mL^−1^	[95]
	polyGGK	SPPS of poly GGK followed by the attachment of diverse sugar moieties through C-linking	TH	From 5 μM to 0.05 nM	[96]
	polyGGK		IRI	From 5 μM to 0.05 nM	[97]
	*Dc*AFP	SPPS of mutant DcAFP substituting the disulfide bonds for lactam bonds	TH	10 mM	[91]

## Abbreviations

AF(G)Ps	antifreeze (glyco)proteins
TH	thermal hysteresis
IRI	ice recrystallization inhibition
SPPS	solid-phase peptide synthesis
IBS	ice-binding site
PyBOP	benzotriazole-1-yloxytris(pyrrolidino)phosphonium
DMTMM	4-(4-6-dimethoxy-1,3,5-triazin-2-yl)-4-methylmorpholinium
Fmoc	fluorenylmethyloxycarbonyl
FIPA	Fluorescence-based ice plane affinity
TRITC	tetramethylrhodamine
PDB	Protein Data Bank
GFP	green fluorescent protein
